# Neural Control of Healthy Aging in Drosophila Through Perceptive Experiences

**DOI:** 10.1093/geroni/igaa057.2634

**Published:** 2020-12-16

**Authors:** Scott Pletcher

**Affiliations:** University of Michigan, Ann Arbor, Michigan, United States

## Abstract

We will describe how neurosensory circuits relate information about nutrition, danger, and mates to initiate rapid changes health and aging, including evolutionarily conserved neural signaling pathways and molecules that extend organism lifespan. These findings provoke the notion that aging may be considered a complex behavior that is acutely malleable, susceptible to sensory influences, and strictly controlled by coordinated sets of neurons.

